# Reinforcement Learning and Decision Making in Depression in Adolescents and Young Adults: Insights from a New Model of the Probabilistic Reward Task

**DOI:** 10.5334/cpsy.147

**Published:** 2025-12-30

**Authors:** Ziwei Cheng, Amelia D. Moser, Jenna Jones, Christopher D. Schneck, David J. Miklowitz, Daniel G. Dillon, Roselinde H. Kaiser

**Affiliations:** 1 Department of Psychology, University of California Berkeley, Berkeley, CA, United States; 2 Department of Psychology and Neuroscience, University of Colorado Boulder, Boulder, CO, United States; 3 Institute of Cognitive Science, University of Colorado Boulder, Boulder, CO, United States; 4 Department of Psychology, Virginia Polytechnic Institute and State University, Blacksburg, VA, United States; 5 Department of Psychiatry, University of Colorado Anschutz Medical Campus, Anschutz, CO, United States; 6 Department of Psychiatry, University of California Los Angeles, Los Angeles, CA, United States; 7 Center for Depression, Anxiety and Stress Research, McLean Hospital, Belmont, MA, United States; 8 Department of Psychiatry, Harvard Medical School, Boston, MA, United States

**Keywords:** computational modeling, depression, development, reward, decision making

## Abstract

Depression is a prevalent psychiatric condition that commonly emerges in adolescence and young adulthood and is associated with reward processing abnormalities. The Probabilistic Reward Task (PRT) is widely used to investigate the impact of depression on reward processing, but prior studies have not comprehensively addressed the reinforcement learning and decision-making mechanisms involved in the task. In 726 adolescents and young adults with varying levels of depression, we collected PRT data and applied a novel computational model with response-outcome learning and evidence accumulation processes to provide new insights into the cognitive processes implicated in depression. Compared to participants with no history of psychopathology, those with depressive disorders showed reduced impact of learned response values on decision bias toward the more frequently rewarded action. In addition, higher levels of anhedonia were associated with slower evidence accumulation during decision-making. Together, these findings improved our understanding of the reinforcement learning and decision-making mechanisms assessed by the PRT and their associations with depression.

Depression is associated with abnormalities in reward processing (Diekhof et al., 2008; Zald & Treadway, 2017). Prior research evaluating differences in reward behavior related to clinical diagnoses and symptom severity has provided insights into such abnormalities (Rupprechter et al., 2018; Safra et al., 2019), with the goal of translating research findings into treatment recommendations (Admon & Pizzagalli, 2015; Treadway & Zald, 2013). Investigation of reward processing abnormalities in adolescents and young adults with major depression has particularly important clinical implications. Given that adolescence is characterized by both changes in reward sensitivity and elevated risk of mood disorders, examining their relationship during this developmental period may provide insight into the etiology of early-onset depression (Forbes & Dahl, 2012; Luking et al., 2016).

The current study administered a well-known reward assay, the Probabilistic Reward Task (PRT; Pizzagalli et al., 2005), to a large sample of adolescents and young adults (Table 1; n = 726) and used computational modeling to draw inferences about (a) cognitive processes that support PRT performance and (b) the impact of depression on those processes. In the PRT, correct identifications of one (“rich”) stimulus are rewarded more frequently than correct identifications of another (“lean”) stimulus. Because of the reward asymmetry, participants typically develop a response bias such that they make the “rich” response more frequently than the “lean” response, even though the rich and lean stimuli are designed to be ambiguous and are shown with equal frequency. Previous studies have found associations between a blunted rich response bias and depression diagnoses (e.g., Vrieze et al. 2013; Morris et al., 2015), decreased dopaminergic signaling (e.g., Grob et al., 2012; Kaiser et al., 2018), and anhedonia (see Kangas et al., 2022, for review). The PRT has offered valuable insight into behavioral biomarkers associated with reward dysfunction and anhedonia, and consequently it is recognized in the NIH’s RDoC Matrix as a validated measure of reward learning (Insel et al., 2010).

**Table 1:** Demographics and clinical characteristics of the sample.


	M (SD)

	N = 726 n (%)

**Gender**	

Cisgender female	476 (66%)

Cisgender male	225 (31%)

Non-binary, transgender, gender-fluid, or not reported	23 (3.2%)

**Ethnicity**	

Hispanic or Latino/Latinx/Latine	122 (17%)

Non-Hispanic and non-Latino/Latinx/Latine	602 (83%)

Not reported	2 (0.28%)

**Race**	

African American	20 (2.8%)

American Indian/Alaskan native	2 (0.3%)

Asian	125 (18%)

Native Hawaiian/other Pacific Islander	1 (0.1%)

White	432 (62%)

More than one race	56 (8.1%)

Other or not reported	62 (8.9%)

**Age**	19 (2)

**Mood Symptoms**	

MASQ-LOI	16.71 (6.79)

	**N = 422** **n (%)**

**Current Mood Diagnoses**	

(Unipolar) Depressive Disorder	121 (29%)

*Major depressive disorder*	*73 (17%)*

*Persistent depressive disorder*	*48 (11%)*

Bipolar Disorder	26 (6.2%)

*Bipolar I disorder*	*11 (2.6%)*

*Bipolar II disorder*	*10 (2.4%)*

*Bipolar disorder not otherwise specified*	*5 (1.2%))*

Non-Psychiatric Control	275 (65%)


*Notes:* MASQ-LOI = Mood and Anxiety Symptom Questionnaire, Loss of Interest Anhedonia subscale.

In addition to behavioral analyses, prior studies have applied analytic techniques to PRT data that can isolate component processes to understand dimensions of reward processing dysfunction in depression (e.g., Pizzagalli et al., 2008; Vrieze et al., 2013; Lawlor et al., 2020; Whitton et al., 2020). In particular, reinforcement learning (RL) models provide a computational perspective on how individuals evaluate and integrate reward information to modify behavior in the PRT. Investigators using the PRT may assume that participants update the values of stimulus-action pairs based on trial-level prediction errors (discrepancy between received and expected reward), such that a bias toward the “rich” response develops over time because of the acquired higher expected value of the rich stimulus. These models have been used to capture the gradual development of response bias across trials and examine relationships between reward learning rate, reward sensitivity, and clinical measures (Huys et al., 2013; Letkiewicz et al., 2022).

However, recent work characterizing decision-making dynamics in PRT performance shows that updates in stimulus-response associations may not be the main driving mechanism in the task (Lawlor et al., 2020; Dillon et al., 2022, 2024). Specifically, studies found that the response bias effect is larger for faster compared with slower response times (RTs). This result suggests that, on many trials, participants prepare to make a rich response very quickly. Thus, participants may learn *response-outcome* associations in the PRT, indicative of a true response bias that does not depend heavily on stimulus evaluation (i.e., participants quickly press “rich” regardless of the stimulus shown; White & Poldrack, 2014). To better understand the underlying decision mechanisms, researchers applied the drift diffusion model (DDM; Ratcliff & Rouder, 1998; Ratcliff & McKoon, 2018; Wiecki et al., 2013) to capture choice frequencies and RT distributions in the PRT (Lawlor et al., 2020; Dillon et al. 2022, 2024; Pitliya et al., 2022; Shen et al., 2024). These analyses revealed a strong starting point bias, suggesting that during the task, participants developed a preference towards the rich response *before* evaluating the stimulus.

Importantly, although both RL models and the DDM have provided insight into reward processing as indexed by the PRT, a recent comparison highlighted shortcomings in both approaches (Dillon et al., 2024). RL models capture learning and development of response bias in the PRT, however, they do not account for RTs (or the dependency of response bias on RTs) and psychometric properties of some parameters are weak. By contrast, the DDM captures decision processes in the PRT and parameters generally have strong psychometric properties, but the DDM does not measure learning and cannot account for the development of response bias, which is central to the PRT. Recently developed integrative models combine RL and DDM modeling frameworks (RLDDM; Pedersen et al., 2017; Pedersen & Frank, 2020), but these existing integrative models do not fit the PRT better than the standard DDM (Dillon et al., 2024). This lack of improvement may stem from two differences between the PRT and traditional RL tasks. First, we hypothesize that in the PRT reward learning is driven by response-outcome (rather than stimulus-response) associations. Second, the PRT requires a difficult perceptual judgment on each trial; the focus on stimulus-locked prediction errors in most RL models, including the RLDDM, may not be ideal for PRT analyses (because the stimuli used in the PRT are too similar to support robust stimulus-locked prediction errors). In short, although much progress has been made, developing models that can better account for learning and decision dynamics in the PRT remains a key goal. Such models may complement information provided by reinforcement learning or decision-making approaches alone, yielding new insights into cognitive and behavioral processes implicated in (and affected by) depression.

To address these goals and to identify processes that may confer risk for future depressive illness, the current study applied an adapted reinforcement learning and drift diffusion model to account for trial-level learning and decision dynamics in the PRT. In the new model (Action-DDM), the learning mechanism is driven by response-outcome associations (the values of response options change based on reward feedback) and the choice mechanism is described by DDM (choices are made by continuously sampling and accumulating noisy evidence over time until a decision boundary is reached). Crucially, the reward learning mechanism influences decision-making such that the value differences between actions modulate (1) drift rate (evidence accumulates more quickly towards boundaries that correspond to actions with higher expected values) and (2) starting point bias (there is a pre-stimulus preference for actions associated with higher expected values).

We evaluated the model by assessing posterior predictive accuracy and parameter recovery, and also tested the split-half reliability of model parameters. We then tested associations between model parameters and depression in the full sample; exploratory analyses repeated these tests in adolescent (ages 13–19) or young adult (ages 20–31) subgroups. The analyses used both a dimensional approach focused on anhedonic depression severity and a categorical approach comparing parameters in individuals diagnosed with depressive disorders and those with no psychiatric history. We hypothesized that individuals currently experiencing more severe anhedonic depression and/or with unipolar depression diagnoses would show reduced reward responsiveness (lower response bias), difficulty learning from rewards (lower learning rate), and slower evidence accumulation during decision-making (lower drift rate).

## Methods

### Participants

A total of 726 participants, ages 13 to 31 years (M = 19.10, SD = 2.44), were recruited from the greater Los Angeles, California, and Boulder, Colorado, areas for one of five research protocols with overlapping procedures (Supplement Table 1–5). The goal was to recruit participants who were in a period of high developmental risk for mood disorders. Eligible participants had to speak fluent English, have normal or corrected-to-normal vision, and report no neurological or cognitive impairments. Sources of recruitment included community flyers, school events, local clinics, and electronic postings. Three of the five protocols aimed to recruit samples with high variance in mood disorders and symptoms, and therefore included eligibility criteria requiring that participants either reported a primary mood disorder, first-degree family history of mood disorder, or had no lifetime history of any psychopathology. Participants were excluded for suicidal ideation when deemed necessary by a study clinician. The research protocols were approved by Institutional Review Boards at the University of California Los Angeles (Protocols #16-001894, #17-000065) and the University of Colorado Boulder (Protocols #18-0600, #18-0415, #19-0130, #20-0475). Legal adults (ages 18 years and older) or guardians of legal minors (ages 17 years and younger) provided informed consent to participate, and minors under 18 also provided assent.

### Procedures

Participants were recruited for an in-person session that included self-report electronic surveys and cognitive tasks administered via computer by trained staff. Participants in three of the five protocols also completed a clinical diagnostic interview (Structured Clinical Interview for DSM-5, SCID-5; First et al., 2015). At the end of the session, participants were debriefed and received financial compensation. The present study used data from the PRT, clinical diagnostic information, and self-reported symptoms of anhedonic depression. Additional research procedures and non-overlapping research results are reported elsewhere (Fassett-Carman et al., 2023; Kaiser et al., 2022; Peterson et al., 2021; Peterson et al., 2022).

### Behavioral Tasks

#### Probabilistic Reward Task (PRT)

**Figure 1:**
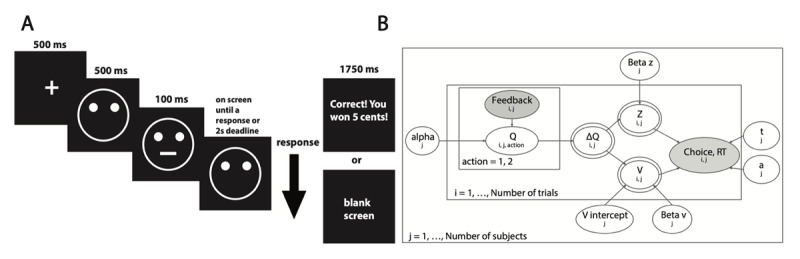
**Probabilistic Reward Task and Graphical Illustration of Action-DDM. (A)** On each trial, participants saw a face with a long or short mouth and responded by pressing a button to indicate which mouth length was shown. Rewards were delivered three times more often for correct identifications of the rich vs. lean stimulus; assignment of short/long mouths to the rich/lean conditions was counterbalanced. **(B)** Graphical illustration of Action-DDM. Shaded nodes represent observed data and unshaded nodes represent parameter estimations. Double-bordered nodes represent trial-wise computed variables. Parameters include *alpha*: learning rate; *B_v_*: the degree to which value differences influenced drift rate; *B_z_*: the degree to which value differences influenced starting point bias; *v_intercept_*: baseline stimulus processing efficiency or drift rate; *t*: non-decision time; *a*: decision threshold.

On each PRT trial (Figure 1A), participants saw a fixation cross (duration: 500 ms), blank cartoon face (500 ms), and a brief presentation (100 ms) of a short (11.5 mm) or long (13 mm) mouth on the face. The cartoon face remained on the screen until participants indicated whether a short or long mouth was shown by pressing the corresponding button on a button box or until the 2 second response deadline was reached. After responding, participants saw either a blank screen (null feedback) or text indicating monetary reward (“Correct! You won 5 cents!”) for 1750 ms. The task consisted of two 100-trial blocks. In each block, an equal number of short and long mouths were presented, but the reinforcement rate was asymmetric: rewards were delivered three times more often for correct identifications of the rich stimulus compared to the lean stimulus (assignment of rich/lean conditions to short/long mouths was counterbalanced across participants). Participants practiced differentiating between the mouth stimuli and were told that not all correct responses would be rewarded, but they were not informed of the asymmetric reinforcement schedule.

### Clinical Measures

#### SCID-5

A subset of the sample (*n* = 422 of 726) were interviewed with the SCID-5 (First et al., 2015). Because the subsample of participants who met criteria for bipolar disorders (BIP: n = 26) was deemed too small for balanced group analyses, diagnostic group comparisons were restricted to participants with current unipolar depressive disorders (UNI: *n* = 121) compared with a non-psychiatric control group (NC: *n* = 275) (see Supplement Table 6 for detailed clinical characteristics).

#### MASQ-Loss of Interest

We evaluated current (past week) anhedonic depressive symptoms with the eight-item Loss of Interest Anhedonia subscale of the Mood and Anxiety Symptom Questionnaire (MASQ; Watson et al., 1995a; 1995b). Participants read items describing anhedonic feelings or experiences and indicated the extent to which the item described how they felt in the past week on a scale from 1 (not at all) to 5 (extremely). Sample items include “Felt withdrawn from others” and “Felt like being alone” (Watson et al., 1995a; 1995b). Responses were summed to yield a measure of anhedonic depression severity.

### Data Analysis

We followed quality control procedures applied in prior PRT studies (Pizzagalli et al., 2005; Lawlor et al., 2020; Dillon et al., 2022; See Supplement for details) After quality control exclusions, PRT data were available for *n* = 671.

#### Performance Statistics

Following prior studies (e.g., Pizzagalli et al., 2005, 2008), our four key measures were accuracy, discriminability, overall response bias, and change in response bias (block 2 minus block 1). Accuracy referred to the number of correct trials divided by the total number of trials, calculated separately for the rich stimulus and lean stimulus. Response bias and discriminability were calculated with the following equations (Pizzagalli et al., 2005):


Equation 1 \[
response~bias=\frac{1}{2}\log \left(\frac{\left(Ric{{h}_{correct}}+0.5 \right)*\left(Lea{{n}_{incorrect}}+0.5 \right)}{\left(Ric{{h}_{incorrect}}+0.5 \right)*\left(Lea{{n}_{correct}}+0.5 \right)} \right)~\]

Equation 2 \[
discriminability=\frac{1}{2}\log \left(\frac{\left(Ric{{h}_{correct}}+0.5 \right)*\left(Lea{{n}_{correct}}+0.5 \right)}{\left(Ric{{h}_{incorrect}}+0.5 \right)*\left(Lea{{n}_{incorrect}}+0.5 \right)} \right)\]

#### Computational Modeling

**Action-DDM**. We adapted an RL model (Huys et al., 2013) and a DDM previously applied to the task (e.g., Lawlor et al., 2020; Dillon et al., 2022) to better characterize learning and decision dynamics in the PRT. In the Action-DDM (Figure 1B), the upper and lower decision boundaries corresponded to “rich” and “lean” responses, respectively. The expected value of response updates according to the delta learning rule (Rescorla and Wagner 1972; Equation 3). On every trial, the model calculated the difference between the expected value of the responses (Equation 4). The speed of evidence accumulation was described by a drift rate intercept (constant across trials and conceptualized as each participant’s baseline stimulus processing efficiency), the action value difference (ΔQ), and a *B_v_* parameter that captured the degree to which the action value difference influenced an agent’s drift rate (Equation 5). In addition, although prior studies of DDMs with RL components assumed that participants could not develop a decision bias (represented as a change in starting point z) because of randomized symbol-value associations (e.g., Pedersen et al., 2017), bias toward the rich response boundary is a key behavioral pattern in the PRT. Therefore, to capture the development of preference toward the rich response as a function of reinforcement history, we included a *B_z_* parameter that described the degree to which the acquired response values influenced an agent’s starting point bias on each trial (Equation 6; *z* is SoftMax-transformed to be bounded between 0 and 1).


Equation 3 \[
\begin{array}{*{20}{c}}
{{Q_{t + 1}}\left( {response} \right) = {Q_t}\left( {response} \right) + \alpha lpha*\left( {r - {Q_t}\left( {response} \right)} \right)}\\
{t = trialnimber,\ response = chosen\ action,\ r = 1\ when\ rewarded\ and\ 0\ otherwise}
\end{array}
\]

Equation 4 \[
\Delta {{Q}_{t}}={{Q}_{rich~response}}-{{Q}_{lean~response}}\]

Equation 5 \[
{{v}_{t}}={{v}_{intercept}}+\Delta {{Q}_{t}}*{{B}_{v}}\]

Equation 6 \[
{{z}_{t}}=\frac{{{e}^{{{B}_{z}}*{{Q}_{t}}\left(rich~response \right)}}}{{{e}^{{{B}_{z}}*{{Q}_{t}}\left(rich~response \right)}}+~{{e}^{{{B}_{z}}*{{Q}_{t}}\left(lean~response \right)}}}\]

Conceptually, the model implies that at the start of the task (ΔQ*_t_* = 0, *v_t_* = *v_intercept_, z_t_* = 0.5), participants perceive the mouth shown on screen and accumulate perceptual evidence until there is sufficient evidence to cross a response threshold. As the trials continue, participants update the expected value of the actions. As Δ*Q_t_* increases (i.e., *Q_rich response_* > *Q_lean response_*), drift rate toward the (correct) “rich” response becomes higher than drift rate for the (correct) “lean” response, leading to higher rich accuracy. In addition, as Δ*Q_t_* increases, the starting point bias shifts towards the rich boundary, leading to faster “rich” responses regardless of the stimulus.

On each trial, we calculated the likelihood of the observed response and RT with the Wiener first-passage time (WFPT) distribution (Wabersich & Vandekerckhove, 2014). For a rich (lean) stimulus, the *v_t_* was positively (negatively) signed to represent evidence accumulation toward the rich (lean) response, and *z_t_* specified the pre-stimulus bias toward the rich response. The decision threshold (*a*) indicated how far apart the two response boundaries were and non-decision time (*t*) accounted for non-decisional processes that added to the RT (i.e., perception and response execution). We applied hierarchical Bayesian modeling to improve parameter estimations at the individual level, and used the RStan package (Stan Development Team, 2022) for posterior sampling.

**Model Convergence**. Models were fit using Markov Chain Monte Carlo sampling. For each model, we drew samples from the posterior distribution three times (10,000 samples, 5,000 burn-in, every fifth sample retained) and computed the Gelman Rubin statistic *R̂* (Gelman & Rubin, 1992) to ensure that the samples converged on a stable solution. *R̂* values compared between- to within-chain variance.

**Model Comparisons and Validations**. See Supplement for detailed alternative model specifications and comparisons. To validate the winning models, we ran posterior predictive checks by using the obtained model parameters to simulate 20 datasets per participant with 200 trials per set; we then compared the simulated results to the observed data.

#### Depression-Related Differences in Behavior

Two-sample *t*-tests compared individuals with unipolar depressive disorders vs. individuals with no psychiatric history for the following parameters: discriminability, response bias, response bias change, *v_intercept_*, B*_v_*, B*_z_, αlpha, a*, and *t*. To assess associations between anhedonic depression severity and reward processing, we computed correlations between the above parameters and scores on the MASQ Loss of Interest scale.

To further examine depression-related differences in PRT within developmental subgroups, we divided participants into younger vs. older groups using a median split and repeated the analyses. Sample 1 (ages 13–19) included 393 participants (198 NC, 67 UNI), and Sample 2 (ages 20–31) included 273 participants (55 NC, 44 UNI).

#### Parameter Validation

To evaluate internal consistency, we split the PRT data by block, fitted the winning model to each block, and quantified across-block parameter agreement with Spearman-Brown correlation coefficients (Brown, 1910; Spearman, 1910). We performed parameter recovery by sampling from the posterior distributions of parameter estimates for each participant, simulating data, fitting the model to the simulated data, and calculating the correlations between the true and recovered parameters.

## Results

### Performance Statistics

Overall, participants developed a bias to choose the rich stimulus and showed better accuracy and shorter RTs on rich compared to lean trials (Table 2). Considering that the stimulus effect on accuracy may vary based on RT (Dillon et al., 2022; Lawlor et al., 2020), we classified each participant’s fast (slow) RT type as the first 0.1 (last 0.9) quantile of their RT distribution (see Figure S1 for RT visualizations). Results from a multilevel logistic regression model to predict trial-level accuracy revealed a main effect of the rich stimulus (B = 0.39, *p* <.001), a main effect of slow RTs (B = 0.83, p <.001) and an interaction between stimulus type and RT type (B = –0.08, *p* = .035). Consistent with prior work, this interaction effect revealed that the rich > lean accuracy effect was larger in trials with fast vs. slow RTs, confirming that the PRT elicited a true response bias (i.e., the rich > lean difference in accuracy was greater on fast trials).

**Table 2:** Summary of PRT performance statistics.


	VARIABLE	MEAN	SD

Block 1	response bias	0.05	0.21

	discriminability	0.67	0.27

	rich acc	0.82	0.10

	lean acc	0.79	0.12

	rich RT	529.64	91.94

	lean RT	530.00	92.43

	rich correct RT	530.67	89.50

	lean correct RT	529.14	89.91

	rich error RT	540.25	143.50

	lean error RT	555.30	144.90

Block 2	response bias	0.08	0.23

	discriminability	0.64	0.27

	rich acc	0.82	0.11

	lean acc	0.76	0.14

	rich RT	532.88	94.20

	lean RT	541.15	95.56

	rich correct RT	532.62	91.94

	lean correct RT	543.75	93.81

	rich error RT	550.44	140.57

	lean error RT	554.92	135.46

Average	response bias	0.07	0.19

	discriminability	0.66	0.25

	rich acc	0.82	0.09

	lean acc	0.77	0.12

	rich RT	531.26	88.70

	lean RT	535.57	88.98

	rich correct RT	531.65	86.19

	lean correct RT	536.45	86.99

	rich error RT	544.77	128.20

	lean error RT	554.31	128.11


### Computational Modeling

**Figure 2:**
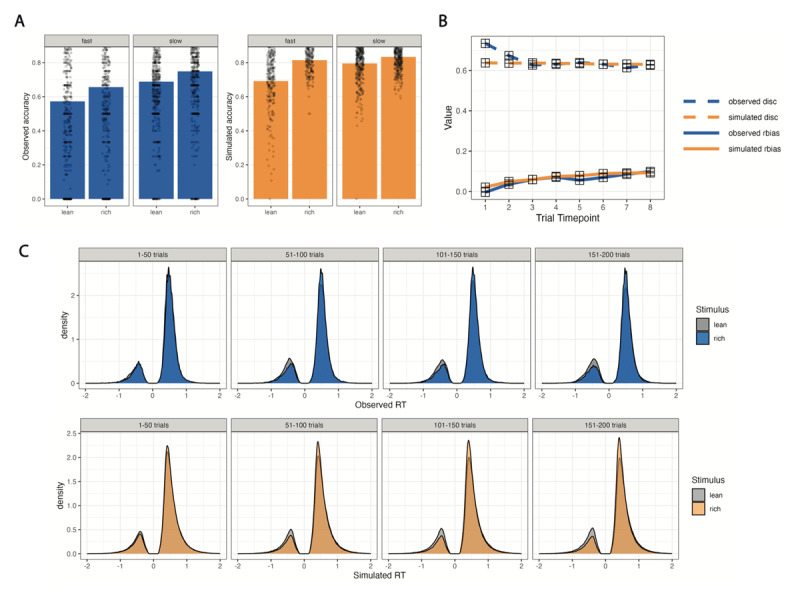
**Posterior Predictive Performance. (A)** Observed and simulated overall accuracy by stimulus type and response time (fast RT < .1 RT quantile; slow RTs > .9 RT quantile). **(B)** Observed and simulated changes in response bias and discriminability. The task trials were binned into eight timepoints with 25 trials each and response bias and discriminability were calculated independently for each timepoint. **(C)** Observed and simulated changes in response time distributions across four timepoints with 50 trials each. For a stimulus (rich or lean), we plotted the RT distributions when participants responded correctly (positively-valued RTs) and incorrectly (flipped to be negatively-valued RTs for illustrations).

The across-run maximum Gelman-Rubin statistic for the MCMC sampler was 1.02, under the recommended threshold of 1.1 (Gelman & Rubin, 1992). The Action-DDM outperformed: (1) a model in which action value differences did not influence drift rate; (2) a model in which action value differences did not influence starting point bias; (3) a model in which the influence of action value differences on drift rate was not controlled by *B_v_*; (4) a model in which the influence of action value differences on starting point bias was not controlled by *B_z_*; and (5) a model with one *B* that controlled the extent to which action value differences influenced both starting point bias and drift rate (see Supplement for alternative model details). The behavior simulated by the Action-DDM captured key patterns in participants’ choices and response times: it captured larger response bias effects for faster compared to slower RTs, a gradual increase in response bias across trials, and the development of faster RTs when choosing the more frequently rewarded action across trials (Figure 2).

### Depression-Related Differences in Behavior

**Figure 3:**
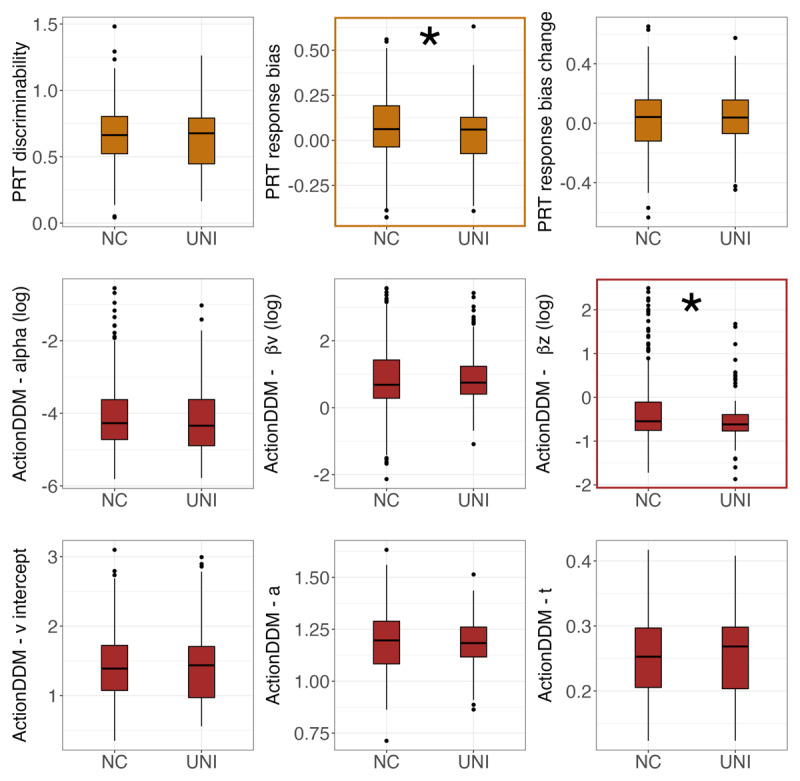
**Comparisons of PRT behavioral parameters between non-psychiatric control group (NC) and unipolar depression group (UNI)**. *Note*: PRT summary statistics and Action-DDM parameters are indicated in gold and red respectively.

**Figure 4:**
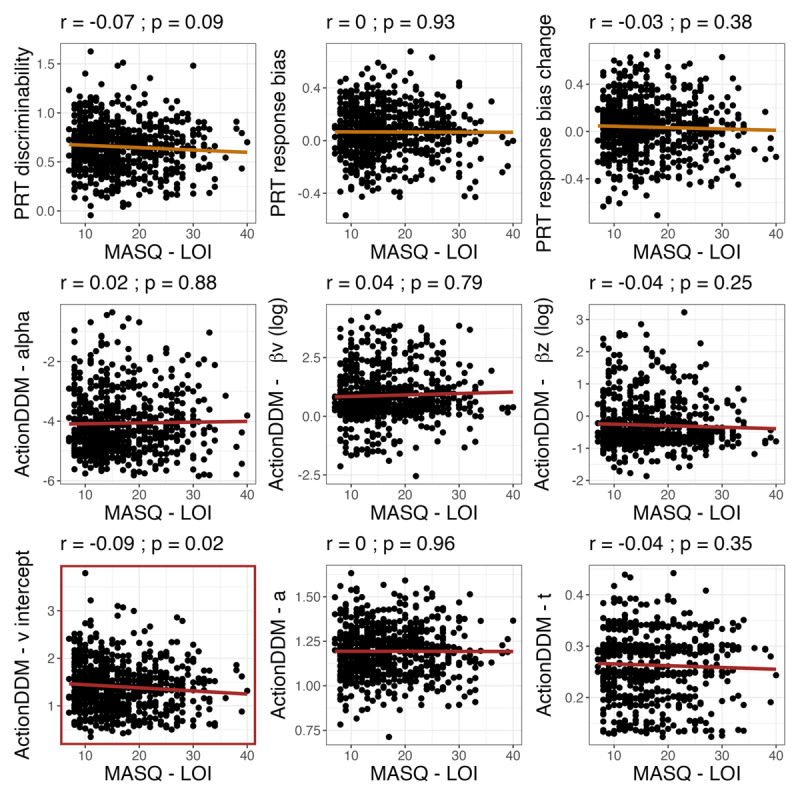
**Correlations between anhedonic symptoms and PRT parameters**. *Note*: PRT summary statistics and Action-DDM parameters are indicated in gold and red respectively.

Compared to controls with no history of psychopathology, depressed individuals showed lower overall response bias (t = –1.99, *p* = .048) and reduced adjustments in starting point bias based on learned response values (B*_z_*, t = –2.51, *p* = .01). There were no significant group differences on other measures or parameters (Figure 3). Severity of anhedonic symptoms was negatively correlated with drift rate intercept (*v_intercept_*; r = –0.09, *p* = .02). There were no other associations between PRT measures or parameters and anhedonic symptom severity (Figure 4). These findings were consistent after controlling for age (see Supplement for detailed analyses on associations between age and task parameters).

Exploratory subgroup analyses using a median split by age found that depressed adolescents (Sample 1) showed trending lower response bias (t = –1.67, *p* = .097), trending lower reward learning rate (*alpha*, t = –1.67, *p* = .098), and reduced adjustments in starting point bias (B*_z_*, t = –2.27, *p* =.02) compared to control adolescents with no history of psychopathology. Among young adults (Sample 2), severity of anhedonic symptoms was negatively correlated with adjustments in starting point bias (B*_z_*; *r* = –0.12, *p* = .04) and no group-level differences emerged. In both samples, higher anhedonia severity was marginally associated with lower drift-rate intercept (r_sample1_ = –0.09, r_sample2_ = –0.10, ps < .10).

### Parameter Validation

Analyses calculated block1-block2 Spearman-Brown coefficients to quantify split-half parameter reliability. For PRT performance measures, Spearman-Brown coefficients were 0.61 (response bias), 0.81 (discriminability), and 0.82 (accuracy). For Action-DDM model parameters, parameter reliability was moderate for learning rate (r_sb_ = 0.65), B*_v_* (r_sb_ = 0.46), and B*_z_* (r_sb_ = 0.67), and strong for *v_intercept_* (r_sb_ = 0.85), decision threshold (r_sb_ = 0.74), and non-decision time (r_sb_ = 0.86). Intraclass correlations yielded comparable reliability estimates: 0.64 (learning rate), 0.35 (B*_v_*), 0.64 (B*_z_*), 0.86 (*v_intercept_*), 0.75 (decision threshold), 0.88 (non-decision time).

Parameter recovery was excellent for *v_intercept_* (r = 0.95), decision threshold (r = 0.95), and non-decision time (r = 0.99), and adequate for learning rate (r = 0.48), B*_v_* (r = 0.52), and B*_z_* (r = 0.54).

## Discussion

This study developed and applied a new computational model to examine dimensions of reward processing using the PRT, and also tested associations between reward parameters and the diagnosis of depression or the severity of anhedonic symptoms. The model successfully captured response bias in both choices and response times, and provided a more integrated account of learning and decision-making processes in the PRT. Youth diagnosed with unipolar depression (especially adolescents) were characterized by blunted biases towards reward, evident in lower response biases and reduced influence of reward values on the starting point bias during decision making. In addition, adolescents and young adults with more severe anhedonia showed slower evidence accumulation. Together, these findings provide insights into the dynamics of learning and decision making in the PRT, and identify distinctive abnormalities associated with depression diagnoses and anhedonia severity.

The finding of reduced response bias, especially among adolescents, replicates past results that demonstrated deficits in reward responsiveness in youth with psychiatric disorders (Morris et al., 2015; Shen et al., 2024). Although recent studies have found that depression-related differences in response bias may not always emerge, they have nonetheless revealed differences in the underlying decision-making parameters (e.g., Lawlor et al., 2020; Dillon et al., 2022, 2024). Importantly, our computational model captured both reward learning and decision-making processes in the task, allowing us to more precisely examine how depression may differentially affect each component. The computational analysis revealed that reward hyposensitivity reflected weaker trial-by-trial adjustments to starting point bias. Such reward hyposensitivity was evident among adolescents diagnosed with unipolar depression, and transdiagnostically associated with higher anhedonic symptoms among young adults. Therefore, all youth learned from feedback and updated action values during the task, but those *without* depression developed a stronger predisposition toward the rich response, leading to faster and more frequent rich responses (i.e., stronger response bias).

Results also showed that more anhedonic youth showed slower evidence accumulation (drift rate intercept). Given the convergence between drift rate intercept and discriminability (level of task difficulty for an individual; see Supplement), this finding may reflect an association between symptom severity and goal-directed information processing. This interpretation is consistent with prior research linking depression with reduced evidence accumulation efficiency (Pitliya et al., 2022; Shen et al., 2024; Sripada & Weigard, 2021). Notably, we did not observe associations between depression or anhedonia and adjustments in drift rate based on reinforcement history. Therefore, *changes* in evidence accumulation speed based on differences in expected values were similar across individuals, but those reporting elevated anhedonia showed slower *overall* evidence accumulation.

Together, findings provided insight into reward processing abnormalities associated with depression during a developmental period of vulnerability to mood disorders. Results suggest that some forms of reward processing abnormality may be more closely related to diagnostic status, whereas others are more closely related to the severity of anhedonic depression. These findings have significant potential clinical implications, as they may help distinguish distinct profiles of reward processing variations and guide the development and targeting of early intervention programs tailored to specific cognitive profiles (Adams et al., 2016).

### Study Limitations

First, the PRT is well-validated and widely used, but other tasks can capture additional dimensions of reward processing. In particular, previous research that reported slower reward learning in depressed individuals often employed traditional learning paradigms based on visual discrimination instead of signal detection (e.g., bandit tasks; Brown et al., 2021; Dombrovski et al., 2010). In addition, learning processes may also differ depending on the outcome types (Pike & Robinson, 2022); however, the current study cannot parse behavior based on outcome valence since the PRT only delivers rewards. Future studies may apply a battery of tasks to more comprehensively and reliably assess aspects of reinforcement learning affected (or unaffected) by depression. Relatedly, the models assumed a fixed learning rate within an individual, but the cognitive strategies underlying task behavior may evolve across trials (Xiong et al., 2025). Such adaptations may reflect meta-learning mechanisms and future studies may model trial-by-trial fluctuations in learning parameters to better characterize how reinforcement learning processes evolve during the PRT.

Second, the current study yielded effect sizes in the small or small-medium range, consistent with prior work reporting on reward processing anomalies in depression (see systematic reviews of Keren et al., 2018 and Halahakoon et al., 2020). Although modest effect sizes are common in clinical research linking depression to neural (Story et al., 2008; Goldstein-Piekarski et al., 2022), genetic (Wichers et al., 2007; Tyrrell et al., 2019) and behavioral (Walsh et al., 2018; Hobbs et al., 2023) variables, combining information from multiple variables (e.g., polygenic risk scoring) may enhance clinical utility and more robustly predict depressive symptoms (Serretti, 2022). Future research may adopt similar approaches, using computational parameters to generate predictive models with improved clinical utility.

Third, it is unclear whether the observed reward processing abnormalies would fluctuate with depressive episode onsets or recoveries, or over the course of youth development. Exploratory analyses revealed that depressed adolescents showed marginally slower reward learning, an effect not observed in young adults with depression. Reduced adjustments in starting point bias were associated with diagnostic status in adolescents and with symptom severity in young adults. Although developmental hypotheses were beyond scope of the present study, results suggest that by isolating specific processes that contribute to behavior in the PRT, the computational model holds potential for refining early identification of reward processing deficits (Adams et al., 2016; Huys et al., 2016; Hauser et al., 2019). Future longitudinal research should investigate the changes of symptom-related or diagnosis-related reward processing abnormalities over the course of adolescent development.

## Conclusion

This study developed a new computational model of RL and decision making in the PRT, and identified different dimensions of reward processing associated with depression diagnoses and anhedonic symptom severity. Future investigations may apply multi-task approaches to more comprehensively assess how depression affects reward processing and explore how reward processing abnormalities change throughout adolescent development.

## Data Accessibility Statement

With the consent/assent of our subjects, data from study sample 3 are available in the National Institute of Mental Health Data Archive (NDA) collection C3598. Participant permissions for data sharing were not achieved for other samples. Software for data analysis were documented in the methods section.

## Additional File

The additional file for this article can be found as follows:

10.5334/cpsy.147.s1 Supplementary file. Supplementary methods and Supplementary Results.
