# Supplementary material for: Reinforcement Learning and Decision Making in Depression in Adolescents and Young Adults: Insights from a New Model of the Probabilistic Reward Task
Source: Comput Psychiatr. 2025 Dec 30;9(1):268–83. doi: 10.5334/cpsy.147 (PMC12758101; doi:10.5334/cpsy.147)

Reinforcement Learning and Decision-Making in Depression: Insights From a New Model of the Probabilistic Reward Task

Ziwei Cheng[1], Amelia D Moser[2,3,4], Jenna Jones[4], Christopher D. Schneck[5], David J. Miklowitz[6],

Daniel G. Dillon[7,8], Roselinde H. Kaiser[2,3,4]

[1] Department of Psychology, University of California Berkeley, Berkeley, CA, United States
[2] Center for Healthy Mind and Mood, University of Colorado Boulder, Boulder, CO, United States
[3] Department of Psychology and Neuroscience, University of Colorado Boulder, Boulder, CO, United States
[4] Institute of Cognitive Science, University of Colorado Boulder, Boulder, CO, United States
[5] Department of Psychiatry, University of Colorado Anschutz Medical Campus, Anschutz, CO, United States
[6] Department of Psychiatry, University of California Los Angeles, Los Angeles, CA, United States
[7] Center for Depression, Anxiety and Stress Research, McLean Hospital, Belmont, Massachusetts, United States
[8] Department of Psychiatry, Harvard Medical School

Corresponding author: Roselinde H Kaiser Roselinde.Kaiser@colorado.edu
Research on Affective Disorders and Development Laboratory
Muenzinger D244, USB 345
University of Colorado Boulder, Boulder CO 80309

Supplementary Methods

Data Trimming and Outlier Analyses

We followed PRT quality control procedures applied in prior studies (Pizzagalli et al., 2005; Lawlor et al., 2020; Dillon et al., 2022) and removed 3.39% of the trials because the raw response time (RT) was faster than 150 ms or slower than 2500 ms, or because the log-transformed RT was more than three standard deviations away from the participant's mean log-transformed RT for the corresponding trial type (rich or lean). We also excluded data sets in which, in any block, the number of RT outliers was greater than 20, fewer than 20 rewards were delivered on rich trials, fewer than 6 rewards were delivered on lean trials, or where the rich/lean reward ratio was lower than 2. Data from 671 of 726 participants passed these quality control checks and were included in the analyses.

Model Comparisons

We compared the winning model reported in main text to: (1) a model in which action value differences did not influence drift rate; (2) a model in which action value differences did not influence starting point bias; (3) a model in which the influence of action value differences on drift rate was not controlled by B_v; (4) a model in which the influence of action value differences on starting point bias was not controlled by B_z; and (5) a model with one B that controlled the extent to which action value differences influenced both starting point bias and drift rate (Table S7). We used the *loo* package in R (Vehtari et al., 2015) to assess model fit and select the most appropriate candidate model given its complexity based on Expected Log Predictive Density (ELPD) and Widely Applicable Information Criterion (WAIC).

In addition, we fitted a drift diffusion model using the StimCoding tools in the HDDM software package (Wiecki et al., 2013) to model to overall choice frequencies and RT

distributions (no trial-level learning in this model; Dillon et al., 2022, 2024; Lawlor et al., 2020). The HDDM returned four parameters: drift rate, decision threshold, starting point bias, and non-decision time (see Lawlor et al., 2020 for model details). We also fitted a reinforcement learning (RL) model (Belief) using the Emfit toolbox to model the trial-level learning dynamics (no RT analysis in this model; Huys et al., 2013). The Belief model returned 5 parameters: learning rate, reward sensitivity, belief uncertainty, instruction sensitivity, and initial bias (see Huys et al., 2013 for model details). We ran posterior predictive checks for the HDDM and Belief models, and compared their performance in explaining learning or decision making processes in the PRT.

Parameter Interpretability

To examine interpretability, we quantified multicollinearity between the parameters in Action-DDM with variance inflation factors (VIF) and tested associations between the parameters and standard PRT performance measures. We also examined linear and quadratic associations between age and the parameters in Action-DDM.

Associations Between Age and Task Parameters

We further tested associations between age and task parameters, to explore age-related change in performance or computational parameters.

Supplementary Results

PRT Model Comparisons

We compared each model's expected predictive accuracy and goodness of fit (Table S7), and identified the Action-DDM (as reported in the main text) as the winning model compared to other model variants.

In addition, we fitted a DDM without trial-level learning (HDDM) and an RL model without including the RT distributions (Belief). The HDDM captured the overall RT distribution

well, while the Belief model captured changes in subjects' performance over time and the development of a behavioral preference toward the rich response through learning. The fitted Action-DDM combined strengths from both models and captured the development of response bias, RT distributions, and interaction between stimulus type and fast vs. slow RT (Figure S2).

Parameter Interpretability

Parameters from Action-DDM showed limited colinearity ($VIF_{Action\text{-}DDM}$ =1.47). Multivariate regression analyses testing associations between PRT performance measures and computational parameters showed that, among the six parameters, discriminability had the strongest association with drift rate intercept (B = 0.88, p <.001). Respone bias had the strongest association with learning rate ($alpha$; B = 0.49, p <.001), influence of value differences on drift rate (B_v; B = 0.75, p <.001), and influence of value differences on starting point bias (B_z; B = 0.36, p <.001).

Associations Between Age and Task Parameters

We found that as age increased, learning rate decreased (r = -0.05, p = .04), non-decision time increased (r = 0.24, p < .001), and response bias marginally decreased (r = -0.07, p = .05; no quadratic association between age and these parameters). No associations were found between age and the influence of value differences on drift rate (B_v) or on starting point bias (B_z) (*ps* > .24). Analyses including quadratic effects revealed that age was linked to increased drift rate, decreased decision threshold, and increased total rewards in the task during adolescence and young adulthood (ps < .05), and these effects stabilized in mid-adulthood (ps < .05). These patterns of age-related differences in key decision-making parameters (e.g., drift rate, threshold) converged with prior studies (Ratcliff et al., 2012; von Krause et al., 2022). Collectively, the results suggest that as age increased from adolescents to young adulthood, participants relied less

on response-outcome learning, required less evidence before making a response (reduced decision threshold), and were better at discriminating between the stimuli at baseline (improved drift rate intercept) to accumulate more rewards in the task; these effects stabilized during mid-adulthood in this sample.

Depression-related differences in behavior as reported in main text remained robust after controlling for age: depressed individuals showed lower overall response bias ($F(1, 361) = 3.98$, $p = .047$) and reduced influence of action value differences on starting point bias ($F(1, 361) = 4.95$, $p = .02$); severity of anhedonic symptoms was negatively correlated with drift rate intercept ($B = -0.09$, $p = 0.02$).

Supplementary References

Dillon, D. G., Lazarov, A., Dolan, S., Bar-Haim, Y., Pizzagalli, D. A., & Schneier, F. R. (2022). Fast evidence accumulation in social anxiety disorder enhances decision making in a probabilistic reward task. *Emotion*, *22*(1), 1–18. https://doi.org/10.1037/emo0001053

Dillon, D. G., Belleau, E. L., Origlio, J., McKee, M., Jahan, A., Meyer, A., Souther, M. K., Brunner, D., Kuhn, M., Ang, Y. S., Cusin, C., Fava, M., & Pizzagalli, D. A. (2024). Using Drift Diffusion and RL Models to Disentangle Effects of Depression On Decision-Making vs. Learning in the Probabilistic Reward Task. *Computational psychiatry (Cambridge, Mass.)*, *8*(1), 46–69. https://doi.org/10.5334/cpsy.108

Huys, Q. J., Pizzagalli, D. A., Bogdan, R., & Dayan, P. (2013). Mapping anhedonia onto reinforcement learning: a behavioural meta-analysis. *Biology of mood & anxiety disorders*, *3*(1), 1-16. https://doi.org/10.1186/2045-5380-3-12

Lawlor, V. M., Webb, C. A., Wiecki, T. V., Frank, M. J., Trivedi, M., Pizzagalli, D. A., & Dillon, D. G. (2020). Dissecting the impact of depression on decision-making. *Psychological medicine*, *50*(10), 1613-1622. https://doi.org/10.1017/S0033291719001570

Pizzagalli, D. A., Jahn, A. L., & O'Shea, J. P. (2005). Toward an objective characterization of an anhedonic phenotype: A signal-detection approach. *Biological Psychiatry, 57*(4), 319–327. https://doi.org/10 .1016/j.biopsych.2004.11.026

Vehtari, A., Gelman, A., & Gabry, J. (2017). Practical Bayesian model evaluation using leave-one-out cross-validation and WAIC. *Statistics and computing*, *27*, 1413-1432.

Wiecki, T. V., Sofer, I., & Frank, M. J. (2013). HDDM: Hierarchical Bayesian estimation of the drift-diffusion model in Python. *Frontiers in neuroinformatics*, *7*, 55610.

Supplementary Tables

Table S1: Demographics of the sample in Study Protocol 1.

	Protocol 1
Protocol Site	UCLA, N = 216[1]
**Gender**	
Female	160 (74%)
Male	55 (25%)
Other	1 (0.5%)
**Age**	20.36 (1.88)
**Ethnicity**	
Hispanic or Latino	49 (23%)
Non-Hispanic and non-Latino	167 (77%)
Other	0
**Race**	
African American	9 (4.2%)
American Indian/Alaskan native	2 (0.9%)
Asian	78 (36%)
More than one race	27 (12%)
Native Hawaiian/other Pacific Islander	1 (0.5%)
White	64 (30%)
Other	35 (16%)
**Annual Family Income**	
Less than $10,000	13 (6.0%)
$10,000 - $25,000	20 (9.3%)
$25,000 - $50,000	33 (15%)
$50,000 - $75,000	40 (19%)
$75,000 - $100,000	44 (20%)
More than $100,000	66 (31%)
**Highest Education (attained by any parent)**	
8th grade or less	11 (5.1%)
Some high school	9 (4.2%)
Finished high school	29 (13%)
Completed GED	2 (0.9%)
Vocational/trade/business school	2 (0.9%)
Some college or 2 year degree	20 (9.3%)
Finished 4 year degree	58 (27%)
Masters degree or equivalent	57 (26%)
Other advanced degree	28 (13%)

Table S2: Demographics of the sample in Study Protocol 2.

	Protocol 2	
Protocol Site	UCLA, N = 154[1]	CU Boulder, N = 64[1]
**Gender**		
Female	106 (69%)	35 (55%)
Male	45 (29%)	25 (39%)
Other	3 (1.9%)	4 (6.2%)
**Age**	20.32 (2.22)	18.58 (2.35)
**Ethnicity**		
Hispanic or Latino	32 (21%)	13 (20%)
Non-Hispanic and non-Latino	122 (79%)	51 (80%)
Other	0	0
**Race**		
African American	5 (3.2%)	1 (1.6%)
American Indian/Alaskan native	0 (0%)	0 (0%)
Asian	31 (20%)	5 (7.8%)
More than one race	24 (16%)	7 (11%)
Native Hawaiian/other Pacific Islander	0 (0%)	0 (0%)
White	79 (51%)	51 (80%)
Other	15 (9.7%)	0 (0%)
**Annual Family Income**		
Less than $10,000	17 (11%)	5 (7.8%)
$10,000 - $25,000	15 (9.7%)	6 (9.4%)
$25,000 - $50,000	21 (14%)	9 (14%)
$50,000 - $75,000	25 (16%)	17 (27%)
$75,000 - $100,000	23 (15%)	12 (19%)
More than $100,000	53 (34%)	15 (23%)
**Highest Education (attained by any parent)**		
8th grade or less	7 (4.5%)	1 (1.6%)
Some high school	6 (3.9%)	1 (1.6%)
Finished high school	8 (5.2%)	1 (1.6%)
Completed GED	2 (1.3%)	0 (0%)
Vocational/trade/business school	1 (0.6%)	1 (1.6%)
Some college or 2 year degree	17 (11%)	12 (19%)
Finished 4 year degree	50 (32%)	14 (22%)
Masters degree or equivalent	38 (25%)	26 (41%)
Other advanced degree	25 (16%)	8 (12%)

Table S3: Demographics of the sample in Study Protocol 3.

	Protocol 3
Protocol Site	CU Boulder, N = 168[1]
**Gender**	
Female	94 (56%)
Male	63 (38%)
Other	11 (6.5%)
**Age**	16.54 (1.88)
**Ethnicity**	
Hispanic or Latino	18 (11%)
Non-Hispanic and non-Latino	150 (89%)
Other	0
**Race**	
African American	2 (1.2%)
American Indian/Alaskan native	0 (0%)
Asian	7 (4.3%)
More than one race	17 (10%)
Native Hawaiian/other Pacific Islander	0 (0%)
White	138 (84%)
Other	0 (0%)
**Annual Family Income**	
Less than $10,000	4 (2.4%)
$10,000 - $25,000	13 (7.9%)
$25,000 - $50,000	22 (13%)
$50,000 - $75,000	29 (18%)
$75,000 - $100,000	34 (21%)
More than $100,000	62 (38%)
**Highest Education (attained by any parent)**	
8th grade or less	0 (0%)
Some high school	0 (0%)
Finished high school	8 (5.0%)
Completed GED	0 (0%)
Vocational/trade/business school	0 (0%)
Some college or 2 year degree	8 (5.0%)
Finished 4 year degree	38 (24%)
Masters degree or equivalent	80 (50%)
Other advanced degree	25 (16%)

Table S4: Demographics of the sample in Study Protocol 4.

	Protocol 4
Protocol Site	CU Boulder, N = 78[1]
**Gender**	
Female	53 (68%)
Male	25 (32%)
Other	0 (0%)
**Age**	19.14 (1.33)
**Ethnicity**	
Hispanic or Latino	8 (10%)
Non-Hispanic and non-Latino	70 (90%)
Other	0
**Race**	
African American	3 (3.8%)
American Indian/Alaskan native	0 (0%)
Asian	2 (2.6%)
More than one race	6 (7.7%)
Native Hawaiian/other Pacific Islander	0 (0%)
White	67 (86%)
Other	0 (0%)
**Annual Family Income**	
Less than $10,000	9 (12%)
$10,000 - $25,000	7 (9.0%)
$25,000 - $50,000	8 (10%)
$50,000 - $75,000	5 (6.4%)
$75,000 - $100,000	10 (13%)
More than $100,000	39 (50%)
**Highest Education (attained by any parent)**	
8th grade or less	0 (0%)
Some high school	0 (0%)
Finished high school	2 (2.6%)
Completed GED	1 (1.3%)
Vocational/trade/business school	1 (1.3%)
Some college or 2 year degree	10 (13%)
Finished 4 year degree	28 (36%)
Masters degree or equivalent	27 (35%)
Other advanced degree	9 (12%)

Table S5: Demographics of the sample in Study Protocol 5.

	Protocol 5
Protocol Site	CU Boulder, N = 46[1]
**Gender**	
Female	28 (61%)
Male	12 (26%)
Other	4 (8.7%)
**Age**	19.12 (0.85)
**Ethnicity**	
Hispanic or Latino	2 (4.3%)
Non-Hispanic and non-Latino	42 (91%)
Other	2 (4.3%)
**Race**	
African American	0 (0%)
American Indian/Alaskan native	0 (0%)
Asian	3 (7.0%)
More than one race	4 (9.3%)
Native Hawaiian/other Pacific Islander	0 (0%)
White	36 (84%)
Other	0 (0%)
**Annual Family Income**	
Less than $10,000	5 (12%)
$10,000 - $25,000	1 (2.3%)
$25,000 - $50,000	4 (9.3%)
$50,000 - $75,000	6 (14%)
$75,000 - $100,000	10 (23%)
More than $100,000	17 (40%)
**Highest Education (attained by any parent)**	
8th grade or less	0 (0%)
Some high school	1 (2.3%)
Finished high school	1 (2.3%)
Completed GED	0 (0%)
Vocational/trade/business school	0 (0%)
Some college or 2 year degree	0 (0%)
Finished 4 year degree	14 (33%)
Masters degree or equivalent	18 (42%)
Other advanced degree	9 (21%)

Table S6: Clinical symptom severity of the sample by study protocol

	Protocol 1	Protocol 2		Protocol 3	Protocol 4	Protocol 5
Protocol Site	UCLA, N = 196	UCLA, N = 153	CU Boulder, N = 63	CU Boulder, N = 164	CU Boulder, N = 69	CU Boulder, N = 42
Symptoms M (SD)						
MASQ-LOI	16.04 (5.44)	19.05 (8.64)	21.59 (6.92)	15.05 (5.78)	14.90 (5.34)	14.16 (5.32)
Mood Diagnoses %						
Unipolar Disorder						
Major depressive disorder	-	19%	46%	5.5%	-	14%
Persistent depressive disorder	-	22%	19%	1.2%	-	0%
Bipolar Disorder						
Bipolar I disorder	-	4.6%	4.8%	0%	-	2.4%
Bipolar II disorder	-	3.3%	4.8%	1.2%	-	0%
Bipolar disorder not otherwise specified	-	1.3%	3.2%	0.61%	-	0%
Non-Psychiatric Control	-	50%	22%	92%	-	83%

Table S7: Model Comparisons among Action-DDM candidate models.

MODEL	DESCRIPTION	ΔELPD	WAIC
ACTION-DDM	$a_{intercept}, t_{intercept}, alpha$ $v_t \sim v_{intercept} + \Delta Q_t * B_v$ $z_t \sim \frac{e^{B_z * Q_t(rich\ response)}}{e^{B_z * Q_t(rich\ response)} + e^{B_z * Q_t(lean\ response)}}$	0	-14097.1
ACTION-DDM_$z_{intercept}$_$beta_v$	$a_{intercept}, t_{intercept}, alpha, z_{intercept}$ $v_t \sim v_{intercept} + \Delta Q_t * B_v$	-477.8	-13141.6
ACTION-DDM_$v_{intercept}$_ $beta_z$	$a_{intercept}, t_{intercept}, alpha, v_{intercept}$ $z_t \sim \frac{e^{B_z * Q_t(rich\ response)}}{e^{B_z * Q_t(rich\ response)} + e^{B_z * Q_t(lean\ response)}}$	-346.2	-13404.8
ACTION-DDM_$beta_v$	$a_{intercept}, t_{intercept}, alpha$ $v_t \sim v_{intercept} + \Delta Q_t * B_v$ $\frac{e^{Q_t(rich\ response)}}{e^{Q_t(rich\ response)} + e^{Q_t(lean\ response)}}$	-84.4	-13928.4
ACTION-DDM_ $beta_z$	$a_{intercept}, t_{intercept}, alpha$ $v_t \sim v_{intercept} + \Delta Q_t$ $z_t \sim \frac{e^{B_z * Q_t(rich\ response)}}{e^{B_z * Q_t(rich\ response)} + e^{B_z * Q_t(lean\ response)}}$	-218.9	-13659.4
ACTION-DDM _$shared$	$a_{intercept}, t_{intercept}, alpha$ $v_t \sim v_{intercept} + \Delta Q_t * B$ $z_t \sim \frac{e^{B * Q_t(rich\ response)}}{e^{B * Q_t(rich\ response)} + e^{B * Q_t(lean\ response)}}$	-276.4	-13582.5

Notes: ELPD refers to expected log predictive density between each model and the winning model (top row). WAIC refers to widely applicable information criterion.

Supplementary Figures

Figure S1. PRT response time and accuracy visualizations by stimulus type. (A) Data points (circle or cross) from bottom to top represent the 0.1, 0.3, 0.5, 0.7, 0.9, and 0.995 response time (RT) quantiles across all participants. Accuracy is higher for the rich vs. lean stimulus when the circles are farther to the right (higher percent correct) than the crosses. The greatest rich > lean accuracy difference was observed in the 0.1 quantile. (B) Fast and slow RTs were operationalized as the first 0.1 and last 0.9 quantile, respectively, of participants' RT distributions. Displayed are the average accuracies with standard errors by trial type (rich versus lean stimuli; fast versus slow response times). As can be seen, the rich > lean accuracy effect is larger for fast vs. slow RTs.

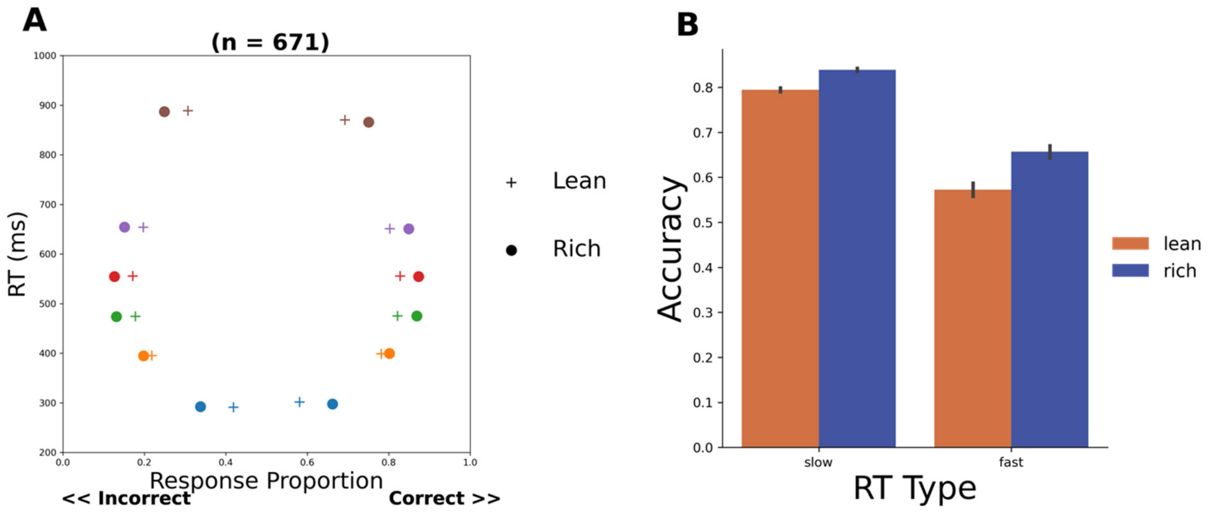

Figure S2. Posterior predictive checks of HDDM and Belief model applied to the PRT. (A) Observed and simulated response time from PRT-HDDM. The PRT-HDDM captured the response time distributions for correct and incorrect (negatively-valued for illustration) responses to lean or rich stimuli, and the pattern that response bias was stronger for faster RTs. However, the model did not include any trial-wise learning or development of the bias across trials. **(B)** Observed and simulated changes in response bias and discriminability from PRT-Belief. The Belief model captured the development of response bias, but did not consider or explain the RT distributions.

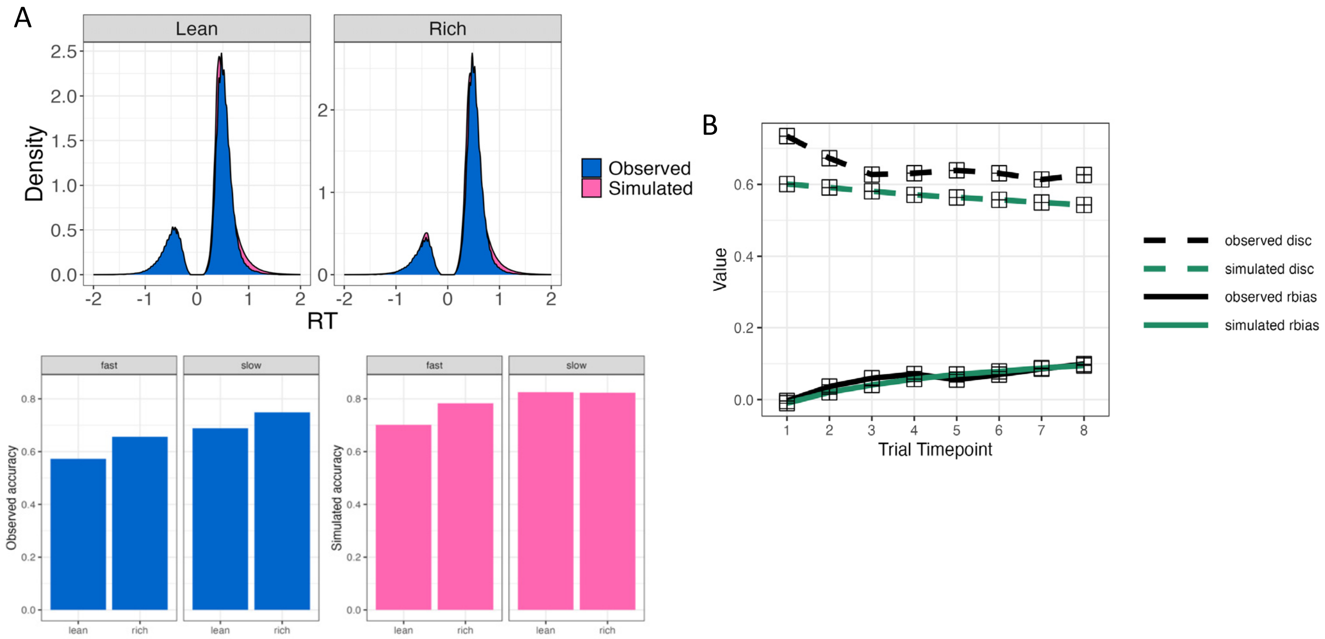

Supplement: Supplementary file. — Supplementary methods and Supplementary Results. [file cpsy-9-1-147-s1.pdf]
